# Increasing Diagnosis of Enamel Hypomineralisation in a Sample of More Than 70 000 Children in the City of Zurich: Repeated Cross‐Sectional Prevalence Analyses and a Retrospective Longitudinal Cohort Analysis

**DOI:** 10.1111/ipd.70110

**Published:** 2026-07-06

**Authors:** Timo Saxer, Safa Gharaviroudsari, Valeria Diener, Daniel Wiedemeier

**Affiliations:** ^1^ Center for Dental Medicine University of Zurich Zurich Switzerland

**Keywords:** hypomineralised second primary molars (HSPM), machine learning in dentistry, molar‐incisor hypomineralisation (MIH), prevalence trends, Switzerland

## Abstract

**Background:**

The prevalence of molar‐incisor hypomineralisation (MIH) has garnered increasing attention, with a likely association to hypomineralised second primary molars (HSPM).

**Aim:**

To elucidate the prevalence of MIH and its association with HSPM.

**Design:**

This retrospective observational study combined repeated cross‐sectional analyses with a retrospective longitudinal cohort analysis of over 70 000 children (851 685 observations) attending public schools in Zurich, Switzerland (2005–2019). Hypomineralised lesions in second primary and first permanent molars were assessed. Prevalence rates and sex differences were calculated using annual age‐specific cross‐sectional samples. Logistic and log‐binomial regressions complemented by machine learning algorithms explored the longitudinal association between HSPM and MIH in children with records both at ages 5–7 and ≥ 8.

**Results:**

HSPM prevalence in five‐year‐olds increased from 5% to 13% between 2005 and 2019. Hypomineralised permanent first molars in eight‐year‐olds increased from 12% to 21%. Children with HSPM were approximately twice as likely to develop hypomineralised permanent molars (OR = 2.3; 95% CI: 2.1–2.5; PR = 1.9; 95% CI: 1.7–2.0).

**Conclusion:**

This large dataset confirms increasing HSPM and MIH diagnosis in Zurich between 2005 and 2019 and supports HSPM as a useful clinical indicator for elevated MIH risk. Limitations include study retrospectivity and potential examiner variability.

## Keypoints

- Large data records show the increase in HSPM and MIH diagnosis between 2005 and 2019 in the city of Zurich.
- When a second primary molar was affected, the likelihood of developing a hypomineralised permanent molar was twice as high.
- The importance of comprehensive health records in data‐driven research on this topic is highlighted.

## 1 Introduction

While dental caries remains a significant oral health challenge in children, recent research has increasingly focused on developmental defects of enamel, such as molar‐incisor hypomineralisation (MIH). Initially mentioned as just secondary findings, idiopathic enamel hypomineralisations on first permanent molars and incisors were first highlighted in an epidemiological study on Swedish children in the late 1970s [1]. In the following decades scientists and clinicians adopted the term to this condition as molar‐incisor hypomineralisation (MIH), as permanent incisors were observed to be concomitantly affected, although usually less severely [2]. The typical clinical findings include demarcated opacities, post eruptive enamel breakdown and hypersensitivities [3, 4]. It is acknowledged, that other types of teeth can be affected by the same condition as well. Particularly hypomineralisations in second primary molars (HSPM) have been well known by paediatric dentists, and several recent studies increasingly focused on it [5, 6, 7, 8, 9, 10, 11]. For the last decades, clinicians and scientists have been in search of the cause of the disease and various hypotheses have been published. A recent meta‐analysis investigated 45 studies in which 36 different aetiologies have been suggested. The authors concluded that systemic and epi−/genetic factors act synergistically resulting in the development of MIH and proposed a multifactorial aetiology [12].

The global awareness and concern about MIH is steadily growing [13]. The worldwide prevalence of MIH differs widely, ranging from 2% to 40% [14]. Several European prospective studies were published in the last few years. The prevalence of MIH in children aged from 6 to 12 years living in northern Poland was at 6% [15]. A study conducted in four German cities found prevalences ranging from 4% to almost 15%, depending on the observed city [11]. A study in Graz, Austria, found MIH present in 7% of the examined children [16]. A recent meta‐analysis, examining temporal trends in MIH prevalence, found no significant evidence. A then performed sub‐analysis of 11 studies, which focused on different age groups, however, suggested strong evidence for an increasing prevalence between 6% (2000) to 14% (2010) [17].

A first prevalence study on MIH in Switzerland has recently been published [18]. MIH was estimated to be 14.8% in school children living in Basel from the age of seven to 15, being in accordance with various European and global studies [11, 14, 19]. Dental health services in Switzerland differ from area to area. In the city of Zurich, dental care for children is organised by the government. Six municipal paediatric dental clinics have been established to provide dental assessments for resident children and adolescents until the age of 18. Regular internal evaluations show that around 85% of Zurich's children are examined annually in this institution (unpublished data provided by the City of Zurich). All public‐school classes from kindergarten (at age four to six) until the end of compulsory education (at age 15–16) attend annual mandatory, but free, check‐ups. Only a limited number of children do not attend the public dental clinics due to the parent's choice to consult a private dentist. The total number of children examined each year, ranging from kindergarten to the end of compulsory education, is around 20 000–30 000 and continues to increase. Using this valuable long‐term record of dental examinations, the following four questions were addressed:

1. What is the prevalence of hypomineralised second primary molars in five‐year‐old children during the period of 2005–2019, and is there a change over time?
2. What is the prevalence of hypomineralised permanent first molars in eight‐year‐old children during the period of 2005–2019, and is there a change over time?
3. Are male and female children equally affected at age five and age eight, respectively?
4. Are children with at least one affected second primary molar more likely to develop hypomineralised permanent first molars?

Thus, this study aimed to evaluate temporal prevalence trends of HSPM and MIH, assess sex differences, and examine whether diagnosed HSPM at ages 5–7 was associated with MIH at age 8 or later. The period from 2005 to 2019 was selected because, since 2005, all dental records are kept digitally, and 2019 was the last year unaffected by the COVID‐19 pandemic, which disrupted data retrieval due to lockdowns. Exploratory statistical tools were used to analyse the large datasets in relation to the first three questions. Likewise, regression and machine learning approaches were employed exploratorily to investigate the fourth question.

## 2 Materials and Methods

### 2.1 Data Acquisition

This retrospective study had two components: repeated cross‐sectional analyses of annual age‐specific prevalence of HSPM and MIH at ages 5 and 8 between 2005 and 2019 (questions 1–3 above) and a retrospective longitudinal cohort analysis of children with records both at ages 5–7 and at age 8 or later (question 4 above).

The study included all children at the age of five or eight who visited a public dental clinic between the years 2005 and 2019. From 2020 onwards, COVID‐19–related restrictions (e.g., cancelled class visits, reduced attendance) substantially disrupted the routine school dental examinations, so post‐pandemic data are not directly comparable with the pre‐COVID records and were not included.

At the age of five, all children attending public school are scheduled for their first mandatory visit. At this point, it is possible to analyse the fully erupted primary dentition. The age of eight was selected as permanent first molars have erupted and can be appropriately assessed. Various authors have suggested focusing on these age groups as well [20, 21, 22]. The analysed teeth in this study were deciduous second molars and permanent first molars. Permanent incisors, known to be regularly affected by hypomineralised lesions, were not included in the analysis as enamel defects could result from trauma during the deciduous dentition [23, 24].

To ensure reliable and sound assessments of a child's oral status, and hypomineralised lesions in particular, regular internal and external trainings are mandatory for every paediatric dentist working in a paediatric dental clinic in Zurich. The trainings include adhering to current clinical guidelines [20]. During each visit, a paediatric dentist assessed the child's oral status. This included the oral hygiene, a stomatologic check, visual caries diagnostics and assessment of the development of the dentition and prevalent malocclusions.

Before examination, a dental assistant instructed the children to brush their teeth with a manual toothbrush. Professional paediatric dentists then conducted the dental checks. The examinations were performed using artificial light, mirrors, probes and if necessary, air driers or cotton rolls. The dental status, particularly the presence of hypomineralised lesions on second primary molars or first permanent molars, was recorded in the patient file to the respective tooth in a dichotomous format (hypomineralisation present: yes/no). The application StomaNet (*Vitodata AG, Oberohringen, Switzerland*) was used for the clinical records. The conduction of the study was approved by the local ethics committee.

### 2.2 Data Processing

Two datasets were extracted from the anonymised digital patient records of the six public dental clinics of Zurich. The first dataset (referred to as *Diagnosed sample*) contained observations from patients diagnosed with at least one hypomineralised lesion, covering the years 2005 to 2019. Each patient's record in this dataset featured key information such as date of birth, sex, the affected teeth and the corresponding clinic visit dates. Rows with missing values for the sex variable were removed from this dataset (16 patients). The second dataset (*Complete sample*) contained information related to every visit made by patients to the six public clinics during the same timeframe. This comprehensive dataset encompassed patients, both diagnosed and undiagnosed with hypomineralised lesions. Rows with non‐positive visit intervals were removed, as they indicated inconsistencies or recording errors (39 observations). In addition, observations with undefined sex were removed (336 observations). Further investigation revealed patients in *Complete sample*, who were not flagged for hypomineralisation but were recorded as diagnosed in *Diagnosed sample*. These observations were removed from both datasets as well. The final dataset *Diagnosed sample* contained 16 797 individuals and the final dataset *Complete sample* contained 71 844 individuals. Taken together, these two cross‐sectional datasets formed the basis to answer the three exploratory, cross‐sectional questions outlined above, as every child observed in a year can be classified as either affected or not affected (statistical unit: patient).

To approach the fourth question, regarding the influence of hypomineralised second primary molars on permanent first molars, a longitudinal dataset was created (*Longitudinal sample*) from the other two datasets. Only patients meeting both of the following two criteria were included:

1. One visit between the age of five and seven: Patients must have at least one recorded visit occurring from the day they turn five years old up until the day they turn seven. Visits within this timeframe are assumed to be related to diagnosing second primary molar hypomineralisation.
2. One visit after the age of eight: Patients must have at least one visit recorded after turning eight years old. Visits at or after eight years of age are assumed to be related to diagnosing hypomineralised first permanent molars.

Children were not included in the *Longitudinal Sample* if no recorded visit was present in one or both required age windows. The *Longitudinal sample* comprised 47 952 individuals with records in both required age windows. In this longitudinal dataset, about 20% of the patients were diagnosed with hypomineralised first permanent molars, resulting in a relatively unbalanced dataset. To potentially improve the performance of the machine learning algorithms, we also created a balanced dataset of this same data using an up‐sampling method (*Upsampled Longitudinal sample*) [25]. The *Upsampled Longitudinal* dataset comprised 76 782 observations.

An overview of the datasets is given in Table 1.

**TABLE 1:** Overview of all four datasets. Note that the *Diagnose sample* contained all children diagnosed with at least one hypomineralised lesion, covering the years 2005 to 2019 in each respective age group. The *Complete sample* then included all children within the same timeframe, that is also the undiagnosed ones and it served as the basis to compute the *Longitudinal sample* and the *Upsampled Longitudinal sample*.

Name of dataset	Description of dataset	Number of individuals	Age group	Diagnosed vs. undiagnosed individuals	Statistical method
Diagnosed sample	All children that were affected at either five or eight years of age	16 797	cf. Figure 1 and Supporting Information	cf. Figure 1 and Supporting Information	Prevalence calculations
Complete sample	All children that were checked at either five or eight years of age	71 844	cf. Figure 1 and Supporting Information	cf. Figure 1 and Supporting Information	Prevalence calculations
Longitudinal sample	All children that were checked at both points in time: between five to seven years of age and at eight years of age or later	47 952	5 to 7 ≥ 8	5735 vs. 42 217 9561 vs. 38 391	Logistic & Log‐binomial regression Elastic Net Regression Random Forest
Upsampled Longitudinal sample	Same as *Longitudinal sample* but artificially upsampled to obtain better properties for the statistical modelling	76 782 (upsampled)	5 to 7 ≥ 8	11 511 vs. 65 271 38 391 vs. 38 391	Logistic & Log‐binomial regression Elastic Net Regression Random Forest

### 2.3 Statistical Analysis

The following analyses were conducted, directly corresponding to our four research questions:

- Questions 1 and 2: Prevalence estimates and corresponding Wilson 95% confidence intervals over time (using *Diagnosed sample* and *Complete sample*),
- Question 3: Sex‐stratified prevalence rates and corresponding Wilson 95% confidence intervals (using *Diagnosed sample* and *Complete sample*),
- Question 4: Risk association between HSPM and MIH (logistic and log‐binomial regression and exploratory machine learning approaches using *Longitudinal sample* and *Upsampled Longitudinal sample*). In this longitudinal analysis, HSPM diagnosis was the exposure, MIH diagnosis the outcome and sex and calendar year were included as covariates.

In particular, annual prevalences (questions 1 and 2) and sex‐stratified prevalences (question 3), together with their 95% confidence intervals, were directly calculated from the two cross‐sectional datasets (*Diagnosed sample* and *Complete sample*).

To assess whether having at least one affected second primary molar increases the likelihood of the development of hypomineralised permanent first molars (question 4), we applied logistic regression on the longitudinal dataset (*Longitudinal sample*) with the explanatory variables of HSPM, sex, the year of the dental check‐up and all potential two‐way interactions of these variables. The model coefficient for HSPM was used to calculate the odds ratio (OR) and its 95% confidence interval. In addition, we estimated the corresponding prevalence ratio (PR) and its 95% confidence interval from a log‐binomial model with the same set of predictors, to provide a more directly interpretable measure of association.

To complement the classical regression approach and account for potential non‐linearities, we employed two machine learning approaches—Elastic Net Regression and Random Forest—on both the original longitudinal dataset (*Longitudinal sample*) and the upsampled longitudinal dataset (*Upsampled Longitudinal sample*). These analyses further explored the occurrence of hypomineralised lesions in permanent molars with regard to the occurrence in the primary molars as well as sex, the year of the dental check‐up and all potential two‐way interactions of these variables. Cross‐validation was applied for model‐validation. The performance of each approach on each dataset was assessed using confusion matrices and ROC curves including AUC measures. Eventually, variable importance tables were computed for each approach. No formal sensitivity analyses were conducted, as the analyses were descriptive and exploratory and relied on routinely collected data after predefined data‐cleaning steps.

All statistical analyses and plots were computed using the software R (*R: A language and environment for statistical computing*. Vienna, Austria: R Core Team (2021)) including the packages, pROC, randomForest, ROSE and caret [25, 26].

## 3 Results

### 3.1 Prevalence of Hypomineralised Second Primary Molars

**FIGURE 1:**
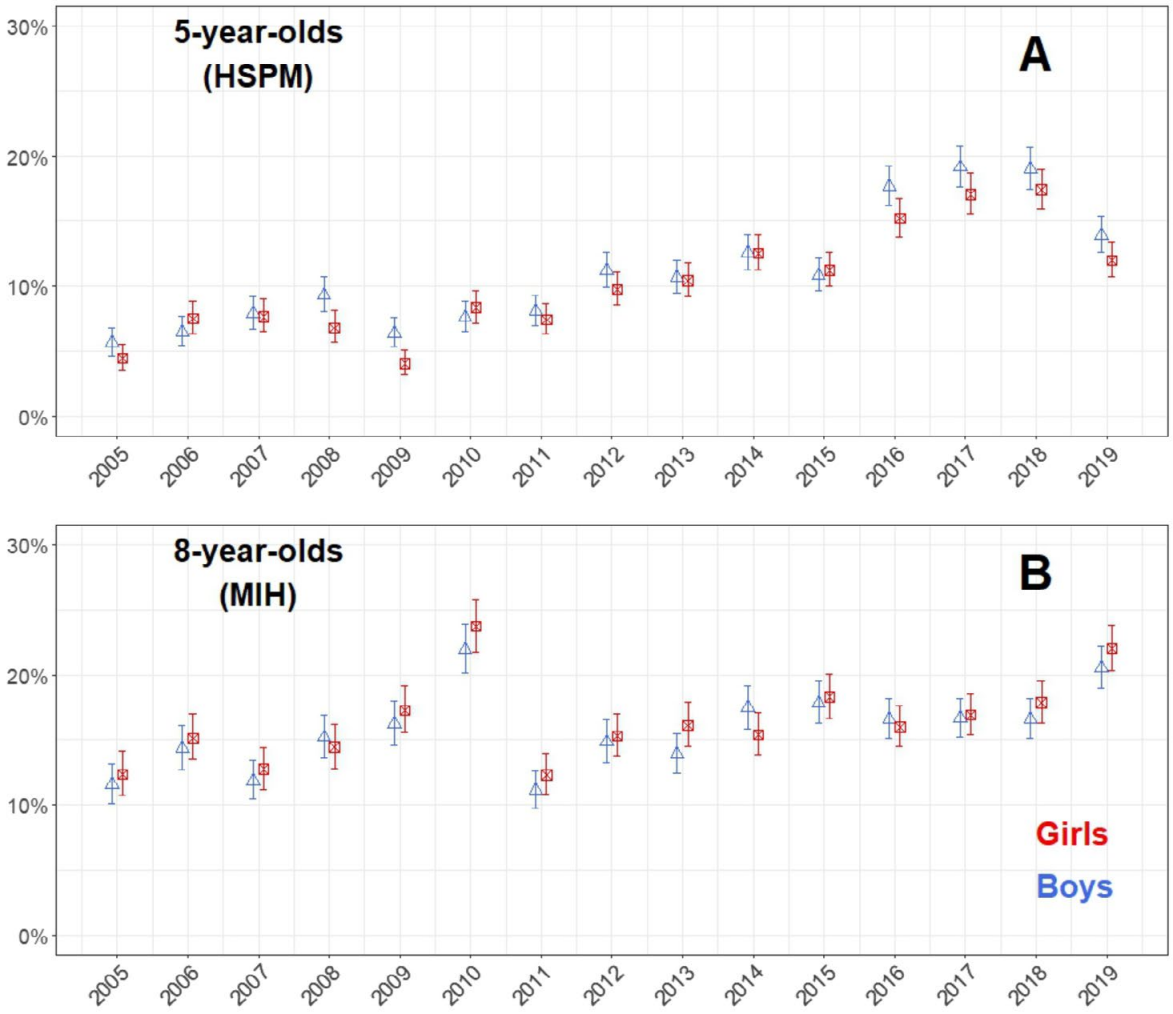
Prevalence rates of hypomineralised second primary molars in five‐year‐olds (A) and hypomineralised first permanent molars in eight‐year‐olds (B) in the city of Zurich, from 2005 to 2019 (data is presented on a yearly basis, error bars indicate Wilson 95% confidence intervals).

The prevalence of the five‐year‐olds diagnosed with at least one hypomineralised second primary molar generally increased over the period from 2005 to 2019. In 2005 the prevalence was 5% and rose to 13% in 2019. The highest prevalence was found in 2017 and 2018 with 18%. (Figure 1A, Supporting Information). Across the years, males appeared to have a slightly higher prevalence of hypomineralised second primary molars than females in Zurich.

### 3.2 Prevalence of Hypomineralised First Permanent Molars

The prevalence of hypomineralised first permanent molars in eight‐year‐olds was generally higher compared to the prevalence of hypomineralised second primary molars in five‐year‐olds during the same period. It also increased over time, although the trend was less pronounced than in the primary dentition. In 2005 the prevalence was 12% and increased to 21% in 2019, with a peak of 23% in 2010. Overall, the difference in prevalence between males and females was less pronounced than for the five‐year‐olds (Figure 1B, Supporting Information).

### 3.3 Association Between Hypomineralised Second Primary Molars and First Permanent Molars

**FIGURE 2:**
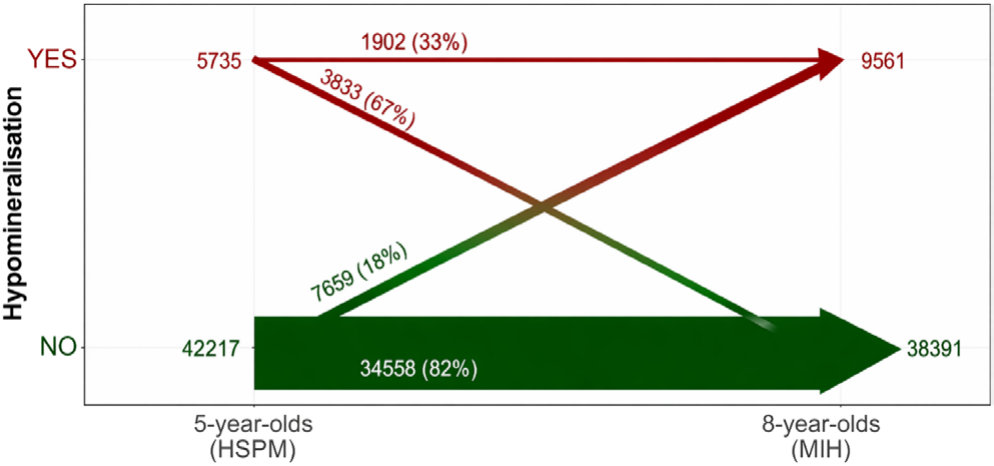
The trajectories show the respective proportions of children in the *Longitudinal sample* data with or without hypomineralised second primary molars developing hypomineralised first permanent molars or remaining unaffected by the condition.

The analysis in our longitudinal sample of around 48 000 children (*Longitudinal sample*) provided further insights into the risk of developing hypomineralised permanent first molars in relation to the occurrence of hypomineralised primary molars. About a third (33%) of affected five‐year‐olds developed hypomineralised permanent molars, compared with less than a fifth (18%) of those children who did not have hypomineralised primary molars (Figure 2). Adjusted for sex, year of the dental check‐up, and their interactions, this corresponded to an odds ratio of 2.3 (95% CI: 2.1–2.5) in the logistic regression and to a prevalence ratio of 1.9 (95% CI: 1.7–2.0) in the log‐binomial regression, suggesting a substantially higher risk for children with hypomineralised primary molars. Nevertheless, the occurrence of hypomineralised lesions in primary molars seemed by no means the sole determinant for permanent molar outcome, as two thirds (67%) of affected children did not develop the condition later.

We thus sought to further investigate the relationship, including the potentially confounding variables of sex and year of the check‐up, using two machine learning approaches on both the original and an upsampled dataset (Table 1). All four resulting models exhibited very low sensitivity and specificity, as evidenced by small AUC's of their respective ROC curves, ranging from 0.57 to 0.59 (95% CI's ranging from 0.56 to 0.61) [26] (Supporting Information). This again indicated that other, unobserved variables likely played an important role in the development of hypomineralised permanent first molars. Rather than indicating a methodological flaw, this low predictive performance underscores the limitations of the current dataset and confirms that even advanced modelling techniques cannot compensate for missing explanatory variables. Regarding the three observed variables and their interactions, the presence of hypomineralised primary molars consistently showed the largest explanatory power across all four models, substantially outperforming the others in variable importance in each of the four models.

## 4 Discussion

This is the first retrospective longitudinal study on prevalence of MIH in Switzerland. Although MIH is a congenital condition, health insurances in Switzerland do not cover potential treatment costs [27, 28], imposing a financial burden on many families. This raises the fundamental question of the prevalence of hypomineralised lesions in the Swiss population and its change over time. In our study the prevalence of primary second molars showed an increase, rising from 5% in 2005 to 13% in 2019, with a peak of 18% in 2017 and 2018. Similar prevalence rates were reported in a cross‐sectional study from Spain (14.5%) and in a prospective study from Brazil (20.1%) [7, 9]. Two prospective studies from India (2015, 5.6%) and from the US (2012, 9.0%) reported slightly lower prevalence rates in primary second molars [5, 8].

The prevalence of hypomineralised permanent first molars increased from 12% in 2005 to 21% in 2019, with a peak in 2010 (23%), showing a generally less pronounced trend. This finding is in most years higher than the global mean of 14.2% and the recently published Swiss prevalence study reporting a prevalence of 14.8% [14, 18]. Similar prevalences were reported in the aforementioned studies from Spain (24.2%) and Brazil (15.7%) [7, 9]. A cross‐sectional study from France reported a prevalence of 18.7% [29]. A recently published meta‐analysis conducted a sub‐analysis of 11 studies with age‐specific prevalences for the assessed children and found a considerable rise in the overall prevalence of MIH from 6% to 14% between 2000 to 2010. Those findings, however, were not based on a meta‐analysis model and require further confirmation. The authors highlighted the need of age‐specific analysis of data, which were incorporated in the present study [17]. One reason for the general increase in diagnosis of HSPM and MIH could be the growing awareness among examining dentists, driven by increased global interest in this topic and the corresponding research on MIH.

Differences between males and females in the prevalence of hypomineralised deciduous second molars were relatively small (≤ 2 percentage points). The same was observed for the prevalences of hypomineralised permanent first molars. These findings are consistent with several other studies from Poland (2019, 5.8% female/6.5% male), Switzerland (2022, 15.5% female/14.1% male) and Turkey (2018, 15% female/13.4% male) that also did not report significant sex differences either [15, 18, 30].

Besides the assessment of prevalences, the question of whether HSPM occurrence is associated with MIH is of considerable interest to clinicians and researchers alike. Due to the overlapping developmental period of second primary molars and first permanent molars, risk factors may similarly disrupt their mineralisation process [5]. A prevalence study alone cannot fully address this question, as only a complete understanding of the underlying biological processes will ultimately provide a comprehensive explanation of the disease. However, the large dataset available provided further statistical insights into the importance of HSPM as an indicator for the development of hypomineralised permanent molars. Among the analysed factors (sex, year of check‐up and HSPM), the presence of hypomineralised primary molars in young years was clearly the strongest predictor of MIH in later years. In fact, our calculations indicated that children with HSPM were approximately twice as likely to develop hypomineralised permanent molars (OR 2.3; 95% CI: 2.1–2.5, PR = 1.9; 95% CI: 1.7–2.0) than children without HSPM.

Our findings for the city of Zurich contribute to the growing body of evidence on the rather strong association between HSPM and MIH. A study conducted in Brazil found that hypomineralised lesions in deciduous teeth resulted in a 33% increase in the prevalence of MIH [31]. Similar findings were reported in other studies, indicating that HSPM can be considered a predictive factor for MIH [5, 8, 9]. Ghanim et al. found that over one third of affected HSPM were associated with hypomineralised first permanent molars [6]. Moreover, a systematic review also concluded that the presence of HSPM is associated with MIH [32]. While all these studies were prospective, the present study analysed data retrospectively.

Collaboration with the public dental clinics in Zurich provided access to a large dataset, and all data were obtained from paediatric dentists. These specialists are aware of MIH and its accurate diagnosis and differentiation from caries, amelogenesis imperfecta and trauma‐related defects. Permanent incisors were excluded from the analysis because of uncertainty regarding the aetiology of the hypomineralised lesions. It is crucial to acknowledge the absence of data from permanent incisors as a limitation of this study. The children's ages of five and eight were selected for data acquisition because full eruption of the analysed teeth could be assumed. However, a measurement bias could not be completely avoided. Due to the retrospective design, the examiners, although trained paediatric dentists, could not be continuously calibrated for clinical examination and the severity of the defects was not recorded. The analysed time‐period covered the earlier years of MIH research, and no official clinical index was used to assess hypomineralised lesions in deciduous or permanent teeth. Because examinations were scheduled within one‐year age bands, some variation in eruption stage and diagnostic visibility is possible, yet full eruption is generally expected, thus any impact on case ascertainment should be small. As our data encompassed a large proportion of children in Zurich (ca. 85%), it likely provides accurate estimates for this population. However, as 15% of children do not attend the annual check‐up, and no information of socio‐economic background, oral health status or access to dental care is available, a sampling bias may be present. Generalisability beyond city boundaries also remains unclear but plausible given comparable findings [9, 11, 15, 18, 31]. Moreover, our analyses are restricted to data up to 2019. The COVID‐19 pandemic markedly altered examination coverage and logistics, such that post‐2020 records cannot be meaningfully combined with the pre‐pandemic series. The present study provides a consistent pre‐COVID baseline. Post‐pandemic trends will require separate analyses. Finally, inclusion of more than just three variables in the statistical modelling of permanent molar hypomineralisation, such as maternal medical conditions and genetic information, could have provided further insights into the development of this condition [33, 34]. Further research using comprehensive health records as well as analytical studies of biochemical processes is needed to fully understand the complex interplay of genetic and systemic factors contributing to this growing oral health concern.

In conclusion, our data indicate that the prevalence of diagnosed hypomineralised second primary molars and hypomineralised first permanent molars increased in Zurich between 2005 to 2019. Males and females were similarly affected, with prevalence estimated at approximately 13% for HSPM and 21% for MIH in 2019. The occurrence of HSPM in a child approximately doubled the likelihood of developing hypomineralised permanent first molars in later years. Large health records, such as the one presented here, will be crucial for future research on this topic, particularly if they incorporate additional potentially important variables.

## Author Contributions

T.S.: conceptualisation (equal); writing – original draft (lead), review and editing (equal). D.W.: conceptualisation (equal); methodology (lead), formal analysis (equal); writing – original draft (supporting), review and editing (equal). V.D.: conceptualisation (equal); writing – review and editing (equal). S.G.: formal analysis (equal); writing – review and editing (equal).

## Funding

The authors have nothing to report.

## Consent

No patient consent was needed to conduct the study.

## Conflicts of Interest

The authors declare no conflicts of interest.

## Supporting information


**Data S1:** ipd70110‐sup‐0001‐Supinfo1.docx.
**Table 1:** The prevalence of five‐year‐old children with at least one hypomineralised second primary molar and eight‐year‐old children with at least one hypomineralised permanent first molar in the city of Zurich from 2005 to 2019, with its corresponding 95% Wilson confidence interval.
**Table 2:** Results of the two employed machine learning algorithms on the longitudinal dataset and upsampled longitudinal dataset.

## Data Availability

The data that support the findings of this study are available on request from the corresponding author. The data are not publicly available due to privacy or ethical restrictions.
