# Supplementary material for: Increasing Diagnosis of Enamel Hypomineralisation in a Sample of More Than 70 000 Children in the City of Zurich: Repeated Cross‐Sectional Prevalence Analyses and a Retrospective Longitudinal Cohort Analysis
Source: Int J Paediatr Dent. 2026 Jul 6;36(5):715–22. doi: 10.1111/ipd.70110 (PMC13446837; doi:10.1111/ipd.70110)
Supplement: Supplementary file 1 [file IPD-36-715-s001.docx]

**Increasing Diagnosis of Enamel Hypomineralisation in a Sample of More Than 70 000 Children in the City of Zurich: Repeated Cross‐Sectional Prevalence Analyses and a Retrospective Longitudinal Cohort Analysis**

**------ Supplementary Information ------**

*Table 1* The prevalence of five-year-old children with at least one hypomineralised second primary molar and eight-year-old children with at least one hypomineralised permanent first molar in the city of Zurich from 2005 to 2019, with its corresponding 95% Wilson confidence interval.

| Year | Affected teeth | Male: affected / total | Prevalence, % (95% CI) | Female: affected / total | Prevalence, % (95% CI) |
| --- | --- | --- | --- | --- | --- |
| 2005 | Primary molars  Permanent molars | 105 / 1855  192 / 1659 | 6 (5 – 7)  12 (10 – 13) | 76 / 1690  185 / 1499 | 5 (4 – 6)  12 (11 – 14) |
| 2006 | Primary molars  Permanent molars | 116 / 1787  236 / 1647 | 7 (05 – 8)  14 (13 – 16) | 125 / 1666  245 / 1617 | 8 (6 – 9)  15 (14 – 17) |
| 2007 | Primary molars  Permanent molars | 143 / 1809  220 / 1851 | 8 (7 – 9)  12 (10 – 13) | 132 / 1720  211 / 1654 | 8 (7 – 9)  13 (11 – 15) |
| 2008 | Primary molars  Permanent molars | 177 / 1901  282 / 1855 | 9 (8 – 11)  15 (14 – 17) | 113 / 1660  244 / 1690 | 7 (6 – 8)  14 (13 – 16) |
| 2009 | Primary molars  Permanent molars | 117 / 1839  290 / 1787 | 6 (5 – 8)  16 (15 – 18) | 75 / 1841  288 / 1666 | 4 (3 – 5)  17 (16 – 19) |
| 2010 | Primary molars  Permanent molars | 153 / 2003  397 / 1809 | 8 (7 – 9)  22 (20 – 24) | 157 / 1878  408 / 1720 | 8 (7 – 10)  24 (23 – 26) |
| 2011 | Primary molars  Permanent molars | 161 / 1998  211 / 1901 | 8 (7 – 09)  11 (10 – 13) | 142 / 1914  204 / 1660 | 7 (6 – 9)  12 (11 – 14) |
| 2012 | Primary molars  Permanent molars | 246 / 2195  273 / 1839 | 11 (10 – 13)  15 (13 – 17) | 201 / 2056  282 / 1841 | 10 (9 – 11)  15 (14 – .17) |
| 2013 | Primary molars  Permanent molars | 244 / 2285  279 / 2003 | 11 (10 – 12)  14 (12 – 16) | 216 / 2069  303 / 1878 | 10 (9 – 12)  16 (15 – 18) |
| 2014 | Primary molars  Permanent molars | 296 / 2349  349 / 1998 | 13 (11 – 14)  18 (16 – 19) | 282 / 2246  295 / 1914 | 13 (11 – 14)  15 (14 – 17) |
| 2015 | Primary molars  Permanent molars | 256 / 2356  392 / 2195 | 11 (10 – 12)  18 (16 – 20) | 250 / 2222  376 / 2056 | 11 (10 – 13)  18 (17 – 20) |
| 2016 | Primary molars  Permanent molars | 425 / 2407  380 / 2285 | 18 (16 – 19)  17 (15 – 18) | 332 / 2184  331 / 2069 | 15 (14 – 17)  16 (15 – 18) |
| 2017 | Primary molars  Permanent molars | 444 / 2319  391 / 2349 | 19 (18 – 21)  17 (15 – 18) | 376 / 2203  380 / 2246 | 17 (16 – 19)  17 (15 – 19) |
| 2018 | Primary molars  Permanent molars | 449 / 2362  391 / 2356 | 19 (18 – 21)  17 (15 – 18) | 401 / 2306  397 / 2222 | 17 (16 – 19)  18 (16 – 20) |
| 2019 | Primary molars  Permanent molars | 337 / 2417  495 / 2407 | 14 (13 – 15)  21 (19 – 22) | 286 / 2380  481 / 2184 | 12 (11 – 13)  22 (20 – 24) |
|  |  |  |  |  |  |

*Table 2* Results of the two employed machine learning algorithms on the longitudinal dataset and upsampled longitudinal dataset.

| **Name of dataset** | **Model** | **Accuracy (95% CI)** | **No Information Rate** | **AUC (95% CI)** |
| --- | --- | --- | --- | --- |
| *Longitudinal sample* | Elastic Net Regression | 0.80 (0.79 – 0.81) | 0.80 | 0.58 (0.56-0.59) |
|  | Random Forest | 0.8 (0.79 – 0.81) | 0.80 | 0.58 (0.56-0.59) |
| *Upsampled longitudinal sample* | Elastic Net Regression | 0.54 (0.53 – 0.55) | 0.50 | 0.57 (0.56-0.58) |
|  | Random Forest | 0.57 (0.56 – 0.58) | 0.50 | 0.59 (0.59-0.60) |
